# Post-dengue subacute thyroiditis in a Peruvian woman: case report and literature review

**DOI:** 10.17843/rpmesp.2024.414.14228

**Published:** 2024-10-15

**Authors:** María de Lourdes Trujillo-Aguirre, Rosa Laurie Marcilla-Truyenque, Juan Eduardo Quiroz-Aldave, Adriana Morales-Moreno, María del Carmen Durand-Vásquez, Marcio José Concepción-Zavaleta, José Paz-Ibarra

**Affiliations:** 1 Endocrinology division, Edgardo Rebagliati Martins National Hospital, Lima, Peru. ">Endocrinology division Edgardo Rebagliati Martins National Hospital Lima Peru; 2 Department of Medicine, Chepén Support Hospital. Chepén, Peru. ">Department of Medicine Chepén Support Hospital Chepén Peru; 3 Infectious Diseases Service, SANNA, El Golf Clinic. Lima, Peru. Infectious Diseases Service SANNA El Golf Clinic Lima Peru; 4 Universidad Científica del Sur, Lima, Peru. Universidad Científica del Sur Universidad Científica del Sur Lima Peru; 5 Department of Medicine. School of Medicine. Universidad Nacional Mayor de San Marcos, Lima, Peru. Universidad Nacional Mayor de San Marcos Department of Medicine. School of Medicine Universidad Nacional Mayor de San Marcos Lima Peru

**Keywords:** Subacute Thyroiditis, Thyrotoxicosis, Scintigraphy, Dengue, Case Reports

## Abstract

Expanded dengue syndrome are unusual conditions, such as subacute thyroiditis (SAT). We present the case of a 38-year-old woman who had dengue without alarm signs for a month, along with cervical pain and increased cervical volume, palpitations, tremor and dysphagia. Hormonal evaluation, ultrasound and thyroid scintigraphy were consistent with SAT. She received corticoids for two months, with remission after four months. SAT is characterized by neck pain, fever and symptoms of thyrotoxicosis. It is associated with viral infections and it comprises a phase of thyrotoxicosis followed by hypothyroidism. Diagnosis involves hormonal and biochemical tests, thyroid ultrasound with Doppler and scintigraphy. This condition is managed with non-steroidal anti-inflammatory drugs and corticosteroids, according to severity. SAT, an infrequent manifestation of dengue, requires a high degree of suspicion and appropriate management. A review of published cases of SAT due to dengue was carried out in the Scopus, PubMed and Web of Science databases, finding six reported cases, mostly in men.

## INTRODUCTION

Dengue is a systemic viral disease caused by the virus of the same name and transmitted by mosquitoes of the genus Aedes. It is currently the leading arthropod-borne disease worldwide ^1^. There has been a notable increase in the number of cases in the last four decades, especially in the Americas and Asia ^2^^,^^3^. In 2024, the World Health Organization (WHO) has reported 7.6 million cases as of April ^4^.

The clinical presentation of dengue varies from asymptomatic, dengue without alarm signs and with alarm signs to severe dengue ^5^. In 2011, WHO included the expanded dengue syndrome, which encompasses a broad spectrum of uncommon presentations of dengue, including gastrointestinal, hepatic, neurological, pulmonary and renal involvement ^6^^,^^7^. Thyroid involvement, in the form of subacute thyroiditis (SAT), is an extremely rare manifestation within this syndrome.

We present the case of a Peruvian female patient diagnosed with dengue without alarm signs, who developed SAT and was treated with prednisone, achieving thyroid function recovery four months later.

## CASE REPORT

A 38-year-old female patient from Sullana, Piura, on the northern coast of Peru, previously healthy, was diagnosed with dengue without alarm signs at a local health center, with positive IgM ELISA test, receiving outpatient symptomatic treatment.

**Figure 1:**
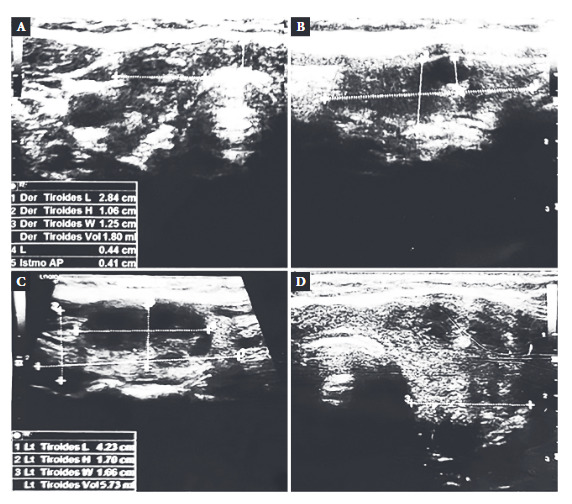
Thyroid ultrasound: (A) Right thyroid lobe, longitudinal and (B) transverse sections show a heterogeneous parenchyma with hypoechogenic areas with poorly defined borders. (C) Left thyroid lobe, longitudinal section shows a predominantly hypoechogenic heterogeneous nodule of 2.78 cm (longitudinal diameter) x 1.5 cm (transverse diameter). (D) Cross section, there is an increase in volume and heterogeneous parenchyma with some poorly defined hypoechogenic areas.

Four weeks later, she presented intense pain in the anterior cervical region radiating to the mandible and auricular region, accompanied by increased volume in that area, general malaise, odynophagia, dysphagia, palpitations and tremor of the limbs. She was evaluated at a local hospital, where a cervical ultrasound showed a thyroid gland with heterogeneous echogenicity, with the presence in the left thyroid lobe of a solid, hypoechogenic nodule with irregular margins, measuring 27x15 mm, with no calcifications, being classified as ACR-TIRADS 4 (American College of Radiology classification). In addition, multiple adenopathies were found in cervical groups II, III and VI left and bilateral supra-sternal, enlarged, rounded, with well-defined contours, homogeneous structure, hyperechogenic lymph node hilum and increased vascular flow, predominantly hilar and central (Figure 1). During the ultrasound, the patient experienced intense cervical pain on contact with the transducer. Due to the finding of a nodule with suspected malignancy, the patient was referred to Lima for further imaging and laboratory studies.

On admission to our hospital, the patient presented palpitations and tremor at rest, compatible with signs of thyrotoxicosis. Thyroid stimulating hormone (TSH) was found at 0.043 IUI/mL, elevated free thyroxine (T4L) and free triiodothyronine (T3L), negative antithyroperoxidase antibodies (ATPO) and antithyroglobulin antibodies (ATG), and elevated thyroglobulin levels (Table 1).

**Table 1:** Evolution of thyroid function test results up to 4-month follow-up.

Test	Admission	2 months	4 months
TSH (NV: 0.4-4 uUI/mL)	0.043	8.81	2.42
T4L (NV: 0.8-1.9)	4.3	0.95	1.37
T3L (NV: 2.2-5.1)	7.7	--	--
ATPO (U/L)	<10	--	--
ATG (U/L)	<20	--	--
Thyroglobulin (NV: 3-40ng/mL)	118.6	--	24.1

ATG: antithyroglobulin antibodies; ATPO: antithyroperoxidase antibodies; T3L: free triiodothyronine; T4L: free thyroxine; TSH: thyroid-stimulating hormone; NV: normal values; TSH: thyroid-stimulating hormone; T3L: free triiodothyronine; T4L: free thyroxine.

**Figure 2:**
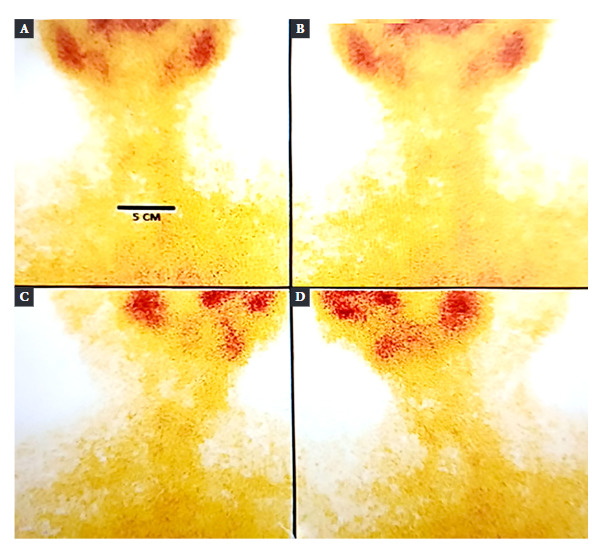
Thyroid scan with Technetium 99m. (A) and (B) Anteroposterior incidences (C) Right anterior oblique incidence (D) Left anterior oblique incidence. It shows physiological uptake at the level of the salivary glands and absence of uptake at the level of the thyroid bed.

Thyroid scintigraphy with Technetium-99m showed absence of radionuclide uptake, compatible with thyroiditis (Figure 2). Due to the ultrasound characteristics of the left thyroid lobe nodule, ultrasound-guided fine needle aspiration biopsy (FNA) was performed, and the result was Bethesda II.

The patient received corticoids for two months, mainly prednisone, with an initial dose of 50 mg/day, which progressively decreased until it was suspended. The patient reported a significant decrease in pain one week after starting corticoid treatment. At one month, she presented a heart rate of 70 beats per minute and the thyroid was palpated (goiter grade Ib) with irregular surface, not painful to palpation and without tremor.

At 2 months, the patient was asymptomatic without corticosteroids, and the results of the CBC showed TSH at 8.81 IUI/mL and free T4 at 0.95 ng/dL. After 2 more months, the patient remained asymptomatic and had TSH at 2.4 IU/mL, free T4 at 1.37 ng/dL, and normal thyroglobulin levels (Table 1).

The patient’s identity has been preserved for this article. We obtained her informed consent and the permission of the chief of the endocrinology service of the Hospital Nacional Edgardo Rebagliati Martins for publication.

## DISCUSSION

In 2024, a major dengue fever outbreak took place in Peru, which had affected more than 250,000 people as of July. This event resulted in the identification of numerous cases with atypical manifestations, such as the present case ^8^. This case corresponds to dengue SAT, which is part of the expanded dengue syndrome, one of the unusual manifestations of severe dengue infection or is associated with underlying host conditions or coinfections ^9^.

SAT, also known as Quervain’s thyroiditis, giant cell thyroiditis, or granulomatous thyroiditis, is the most frequent cause of thyroid pain ^10^^,^^11^. It develops due to inflammatory damage to thyroid follicles, leading to their destruction and dysregulated release of thyroid hormones ^12^. It is characterized by anterior cervical pain, usually accompanied or preceded by fever, as well as systemic symptoms such as fatigue, myalgias and arthralgias ^12^^-^^14^. The pain associated with SAT may radiate to areas such as the parieto-occipital areas, ears, jaw, throat and superior mediastinum. The thyroid gland is usually palpable, elongated and painful, and in most cases mild symptoms of thyrotoxicosis are present ^10^^,^^13^^,^^15^.

SAT has an incidence of two to five cases per 100,000 persons per year ^12^^)^ and mainly affects women between 25 and 50 years of age ^14^. Association has been found with various viral infections, including mumps virus, influenza, rubella, measles, hepatitis E, echovirus, coxsackie, adenovirus, parvovirus B19, orthomyxovirus, human immunodeficiency virus, Epstein-Barr, and SARS-CoV-2, particularly in genetically susceptible individuals (HLA-B35 and HLA-B67 genotypes) in the two to six weeks prior to the onset of SAT symptoms ^12^. Although its association with dengue virus is not well established ^16^, in our case, SAT occurred four weeks after dengue infection.

SAT is a transient, self-limited condition that begins with a phase of thyrotoxicosis lasting three to six weeks, in which TSH is below 0.01 mU/L, and T4L and T3L levels are high or normal ^12^^,^^14^. This stage is followed by a phase of hypothyroidism that can last up to six months ^14^. The latter is due to depletion of reserves and lack of thyroid hormone synthesis ^15^. In SAT, the absence of antithyroid antibodies is typical. However, the presence of ATPO has been reported in 15%, ATG in 30-50%, and even antibodies against the thyrotropin receptor (TRAb) in 6% of cases. This could be due to the release of thyroid antigens due to glandular damage, or reflect a higher prevalence of thyroid autoimmunity ^15^.

Our findings are in line with those described for thyroid hormones and antithyroid antibodies. A characteristic laboratory finding is a very high ESR ^14^, in addition to elevated C-reactive protein (CRP), mild anemia, leukocytosis and elevated thyroglobulin levels ^10^^,^^14^^,^^15^. In our case it was not possible to request CRP and ESR tests, which would have contributed to the diagnosis.

Thyroid FNA is important to differentiate SAT from thyroid cancer or metastasis, since they share ultrasound characteristics, as in this case ^17^. Cytologic features are nonspecific and vary according to the stage of the disease. It is common to observe intra-vacuolar cytoplasmic granules in follicular cells, epitheloid granulomas and multinucleated giant cells in an inflammatory background. As inflammation decreases, fibrosis is found in different degrees with epithelioid granulomas ^14^.

Thyroid ultrasound reveals hypoechoic and irregular areas ^15^, while evaluation of vascularity by color Doppler shows normal or decreased flow ^12^. Although in our case, vascularity evaluation was not performed, some reports of SAT associated with dengue showed increased vascularity ^16^^,^^18^, which could be a differential finding in the context of dengue infection. Iodine-131 or Technetium-99m scintigraphy generally show low or no uptake, as evidenced in our patient ^12^.

The similarity of its symptoms to pharyngitis can lead to misdiagnosis, resulting in the inappropriate use of antibiotics in up to 50% of cases ^15^^,^^16^. Furthermore, differentiating SAT from Graves’ disease is a diagnostic challenge. However, there are useful tools such as platelet to lymphocyte ratio and monocyte to eosinophil ratio, which tend to be higher in SAT cases. A free T4/free T3 ratio greater than 3.33 is found, as well as a total T4/total T3 ratio greater than 0.041 and reduced thyroid artery blood flow ^15^^,^^19^. Misdiagnosis of SAT may delay the recognition of thyroid malignancies, such as metastases, or suppurative thyroiditis, which worsens with glucocorticoid administration, and lead to inappropriate treatment ^14^^,^^17^. There are no strict diagnostic criteria for SAT; for decades the diagnosis has been based on a painful goiter and increased ESR. However, Stasiak and collaborators proposed the diagnostic criteria detailed in Table 2, focused on ruling out malignancy or confirming SAT in cases with inconclusive clinical picture such as absence of pain. They describe that the three main criteria and at least one of the additional criteria are required for an accurate diagnosis ^17^.

**Table 2:** Diagnostic criteria for subacute thyroiditis _^(17^_^)^.

Main criteria
Elevated erythrocyte sedimentation rate (or at least CRP).
Hypoechoic area(s) with blurred margins and decreased vascularity on thyroid ultrasound.
Typical FNA result for subacute thyroiditis or at least exclusion of malignancy.
Additional criteria
Hard lump in the thyroid.
Thyroid pain and sensitivity.
Elevation of serum T4L and suppression of TSH.
Reduced uptake of radioactive iodine.

FNA: fine needle aspiration biopsy; CRP: C-reactive protein; TSH: thyroid-stimulating hormone; ESR: erythrocyte sedimentation rate; T4L: free thyroxine.

Beta-blockers are useful for symptomatic management, while antithyroid drugs are not indicated ^12^^,^^14^. Nonsteroidal anti-inflammatory drugs (NSAIDs) are the first-line treatment for mild to moderate cases, with which patients usually improve in approximately 5 weeks ^10^^,^^12^^,^^14^. Glucocorticoids are considered if symptoms are severe or if the patient does not respond adequately to NSAIDs ^10^^,^^12^ because they provide symptomatic relief in less than 24 hours ^10^. Typically, prednisolone or prednisone at doses of 30-40 mg daily for 1-2 weeks is recommended, followed by a gradual reduction over a six-week period ^10^^,^^12^. However, up to 20% of patients may require treatment for more than eight weeks due to recurrence of symptoms ^10^. It may be necessary to initiate replacement with levothyroxine in the subsequent hypothyroid phase ^14^. To date, there is no definitive treatment protocol for SAT ^10^. One of the challenges in treatment is finding balance between the risk of recurrence by reducing corticosteroids and the complications associated with prolonged therapy ^15^. Approximately 85-90% of patients recover normal thyroid function within one to four months ^12^^,^^14^. The natural evolution of SAT does not depend on the clinic or the treatment used ^10^.

We carried out a review of the cases published to date on SAT due to dengue fever, in the Scopus, PubMed and Web of Science databases, using as keywords: “subacute thyroiditis”, “dengue” and “dengue fever”. We found six cases reported in the literature ^7^^,^^13^^,^^16^^,^^18^^,^^20^^,^^21^, five of which correspond to males, with an average age of 35 years (Table 3). This higher frequency of the male sex is different from that described in general for SAT. From the onset of dengue symptoms, an average of 7.5 days elapsed until the manifestations of SAT, although the period was 4 times longer in our case. Painful cervical swelling, difficulty in swallowing, fever, tachycardia and tremors were found in the vast majority of patients. Exophthalmos was reported in only two cases. Ultrasonography revealed heterogeneous findings including diffuse enlargement, multinodular goiter and complex solid cystic cystic lesion, while Doppler showed increased vascularity. In three cases Technetium-99m scintigraphy showed decreased or absent uptake, and Iodine-131 was used in one case, with the same result. Treatments included corticosteroids, propranolol, NSAIDs and in one case, propylthiouracil due to concomitant thyroid storm. Follow-up was only performed in one case, where the recovery of thyroid function was reported at six weeks.

**Table 3:** Reported cases of SAT associated with dengue.

Author, country, year	Sex	Age (years)	Time (days) *	Symptoms	ESR (mm/h)	TSH (UI/mL)	Ultrasound	Gammagraphy	Treatment
Mangaraj, India, 2021	F	38	10	Painful cervical swelling, fever, tachycardia, tremor	86	0.06	Diffuse elongation, heterogeneous parenchyma. Vascularity: slight increase	No uptake of 99mTc	Propranolol, prednisolone
Bhushan, India, 2018	M	23	5	Painful cervical swelling, tachycardia, exophthalmos	101	0.17	Cystic, solid, complex lesion in the right lobe. Vascularity: increased	Low uptake of 99mTc	Propranolol, steroids, NSAIDs, antibiotics
Dwipayana, Indonesia, 2017	M	33	--	Cervical swelling, fever, tachycardia, exophthalmos, tremor.	--	0.05	Bilateral multinodular goiter	--	Propranolol, methylprednisolone IV pulse, ceftriaxone, propylthiouracil
Mo Z, China, 2016	M	65	6	Not specified	--	0.09	--	--	IV pulse of methylprednisolone
Sheraz, Pakistan, 2016	M	32	--	Painful cervical swelling, difficulty in swallowing, fever, tachycardia, tremor.	43	0.05	--	Low uptake of I131	Propranolol, steroids, ibuprofen
Assir, Pakistan, 2012	M	20	9	Painful cervical swelling, fever, tachycardia, tremor	62	0.01	Vascularity: Decreased	No uptake of 99mTc	Propranolol, prednisone

* Time refers to the interval between the onset of dengue symptoms and the onset of SAT symptoms. 99mTc: Technetium-99m scan; NSAIDs: nonsteroidal anti-inflammatory drugs; IV: intravenous; F: female; I131: Iodine-131 scan; M: male; TSH: thyroid-stimulating hormone; ESR: erythrocyte sedimentation rate.

Limitations of our study included the lack of evaluation of thyroid vascularity by Doppler and the absence of ESR measurement. Nevertheless, hormonal analysis and scintigraphy confirmed the diagnosis.

In conclusion, our report underlines the importance of identifying atypical manifestations of dengue, such as SAT, which is rare and its diagnosis requires a high degree of suspicion, taking into account the clinical, laboratory and epidemiological history, since thyroid scintigraphy is not always available in all settings. In recent years, ultrasound and thyroid FNA have also become important for diagnosis when there is doubt.

## References

[1] Schaefer TJ, Panda PK, Wolford RW (2024). StatPearls.

[2] Paz-Bailey G, Adams LE, Deen J, Anderson KB, Katzelnick LC (2024). Dengue. Lancet.

[3] Guzman MG, Gubler DJ, Izquierdo A, Martinez E, Halstead SB (2016). Dengue infection. Nat Rev Dis Primers.

[4] World Health Organization Dengue - Global situation.

[5] World Health Organization (2009). Dengue: Guidelines for Diagnosis, Treatment, Prevention and Control: New Edition.

[6] World Health Organization (2011). Regional Office for South-East Asia. Comprehensive Guideline for Prevention and Control of Dengue and Dengue Haemorrhagic Fever. Revised and expanded edition.

[7] Mo Z, Dong Y, Chen X, Yao H, Zhang B (2016). Acute transverse myelitis and subacute thyroiditis associated with dengue viral infection A case report and literature review. Exp Ther Med.

[8] Dirección Sub Regional de Salud Luciano Castillo Colonna - Sullana (2024). Sala situacional Dengue Diaria del 16 de Julio del 2024 - OFICINA DE EPIDEMIOLOGÍA.

[9] Mallhi TH, Khan YH, Adnan AS, Tanveer N, Aftab RA (2021). Expanded Dengue Syndrome.

[10] Ray I, D'Souza B, Sarker P, Agarwal P (2022). Management of subacute thyroiditis - a systematic review of current treatment protocols. Int J Gen Med.

[11] Rafiei N, Masoudi M, Jadidi H, Ghaedi A, Jahani N, Ebrahimi S (2023). The association of subacute thyroiditis with viral diseases a comprehensive review of literature. Przegl Epidemiol.

[12] Chaker L, Cooper DS, Walsh JP, Peeters RP (2024). Hyperthyroidism. Lancet.

[13] Assir MZK, Jawa A, Ahmed HI (2012). Expanded dengue syndrome subacute thyroiditis and intracerebral hemorrhage. BMC Infect Dis.

[14] Lanzo N, Patera B, Fazzino GFM, Gallo D, Lai A, Piantanida E (2022). The old and the new in subacute thyroiditis an integrative review. Endocrines.

[15] Stasiak M, Lewinski A (2021). New aspects in the pathogenesis and management of subacute thyroiditis. Rev Endocr Metab Disord.

[16] Bhushan D (2018). Subacute thyroiditis a rare complication of dengue. J Assoc Physicians India.

[17] Stasiak M, Michalak R, Lewinski A (2019). Thyroid primary and metastatic malignant tumours of poor prognosis may mimic subacute thyroiditis - time to change the diagnostic criteria case reports and a review of the literature. BMC Endocr Disord.

[18] Mangaraj S (2021). Subacute thyroiditis complicating dengue fever - case report and brief review of literature. Trop Doct.

[19] Narkar RR, Mishra I, Baliarsinha AK, Choudhury AK (2021). Rapid differential diagnosis of thyrotoxicosis using T3/T4 ratio, FT3/FT4 ratio and color Doppler of thyroid gland. Indian J Endocrinol Metab.

[20] Dwipayana IMP, Nugraha IBA, Semadi S, Wirawan IMS (2017). Thyroid crisis in a toxic multinodular goiter patient triggered by a Den-3 subtype dengue infection. Biomed Pharmacol J.

[21] Sheraz F, Tahir H, Saqi J, Daruwalla V (2016). Dengue fever presenting atypically with viral conjunctivitis and subacute thyroiditis. J Coll Physicians Surg Pak.

