# Supplementary material for: Post-dengue subacute thyroiditis in a Peruvian woman: case report and literature review
Source: Rev Peru Med Exp Salud Publica. 2024 Oct 15;41(4):431–7. doi: 10.17843/rpmesp.2024.414.14228 (PMC11797575; doi:10.17843/rpmesp.2024.414.14228)
Supplement: Translation

# Translation: Tiroiditis subaguda post dengue en una mujer peruana: reporte de caso y revisión de la literatura

**Authors:** María de Lourdes Trujillo-Aguirre, Rosa Laurie Marcilla-Truyenque, Juan Eduardo Quiroz-Aldave, Adriana Morales-Moreno, María del Carmen Durand-Vásquez, Marcio José Concepción-Zavaleta, José Paz-Ibarra

**Affiliations:** 1 División de Endocrinología, Hospital Nacional Edgardo Rebagliati Martins, Lima, Perú.; 2 Servicio de Medicina, Hospital de Apoyo Chepén. Chepén, Perú.; 3 Servicio de Infectología, SANNA, Clínica El Golf. Lima, Perú.; 4 Universidad Científica del Sur, Lima, Perú.; 5 Departamento de Medicina. Facultad de Medicina. Universidad Nacional Mayor de San Marcos, Lima, Perú.

**Keywords:** Tiroiditis Subaguda, Tirotoxicosis, Cintigrafía, Dengue, Informes de Casos

## Abstract

El síndrome de dengue expandido son afectaciones poco habituales, como la tiroiditis subaguda (TSA). Presentamos el caso de una mujer de 38 años quien tuvo dengue sin signos de alarma un mes atrás, presentó dolor y aumento de volumen cervical, palpitaciones, tremor y disfagia. La evaluación hormonal, ecografía y gammagrafía tiroidea fueron consistentes con TSA. Recibió corticoterapia durante dos meses, remitiendo el cuadro a los cuatro meses. La TSA se caracteriza por dolor cervical, fiebre y síntomas de tirotoxicosis. Está asociada con infecciones virales. Comprende una fase de tirotoxicosis seguida de hipotiroidismo. Su diagnóstico involucra exámenes hormonales, bioquímicos, ecografía tiroidea con Doppler y gammagrafía. Se maneja con antiinflamatorios no esteroideos y corticoides, según la severidad. La TSA, una manifestación infrecuente del dengue, requiere un alto grado de sospecha y manejo adecuado. Se realizó una revisión de casos publicados de TSA por dengue, en las bases de datos de Scopus, PubMed y Web of Science, encontrando seis casos reportados, en su mayoría en varones.

## INTRODUCCIÓN

El dengue es una enfermedad viral sistémica causada por el virus homónimo y transmitida por mosquitos del género Aedes. Actualmente, constituye la principal enfermedad transmitida por artrópodos a nivel mundial ^1^. En las últimas cuatro décadas, se ha observado un notable incremento de casos, especialmente en América y Asia ^2^^,^^3^. En el 2024 la Organización Mundial de la Salud (OMS) ha notificado 7,6 millones de casos hasta el mes de abril ^4^.

La presentación clínica del dengue varía desde cuadros asintomáticos, dengue sin signos de alarma y con signos de alarma hasta dengue grave ^5^. En el 2011, la OMS incluyó el síndrome de dengue expandido, que abarca un amplio espectro de presentaciones poco comunes del dengue, incluyendo afectaciones gastrointestinal, hepática, neurológica, pulmonar y renal ^6^^,^^7^. La afectación tiroidea, en forma de tiroiditis subaguda (TSA), es una manifestación extremadamente rara dentro de este síndrome.

Presentamos el caso de una paciente mujer peruana diagnosticada con dengue sin signos de alarma, quien desarrolló TSA y fue tratada con prednisona, logrando la recuperación de la función tiroidea cuatro meses después.

## REPORTE DE CASO

Paciente mujer de 38 años, procedente de Sullana, Piura en la costa norte del Perú, previamente sana, quien fue diagnosticada con dengue sin signos de alarma en un centro de salud local, con prueba de ELISA IgM positiva, recibiendo tratamiento sintomático ambulatorio.

Cuatro semanas después, presentó dolor intenso en la región cervical anterior irradiado a mandíbula y región auricular, acompañado de aumento de volumen en dicha zona, malestar general, odinofagia, disfagia, palpitaciones y temblor de extremidades. Fue evaluada en un hospital de su localidad, donde se le realizó una ecografía cervical que mostró una glándula tiroidea con ecogenicidad heterogénea, con la presencia en el lóbulo tiroideo izquierdo de un nódulo sólido, hipoecogénico, de márgenes irregulares, el cual medía 27x15 mm, no presentaba calcificaciones, siendo catalogado como ACR-TIRADS 4 (clasificación del Colegio Americano de Radiología). Además, se observaron múltiples adenopatías en los grupos cervicales II, III y VI izquierdos y supraesternales bilaterales, aumentadas de tamaño, redondeadas, con contornos bien definidos, estructura homogénea, hilio ganglionar hiperecogénico y flujo vascular aumentado, de predominio hiliar y central (Figura 1). Durante la ecografía, la paciente experimentó dolor cervical intenso, al contacto con el transductor. Debido al hallazgo de un nódulo con sospecha de malignidad, la paciente fue referida a Lima para completar con los estudios de imágenes y laboratorio.

**Figura 1:** Ecografía de tiroides. (A) Lóbulo tiroideo derecho, en los cortes longitudinal y (B) transversal se muestra un parénquima heterogéneo con zonas hipoecogénicas con bordes mal definidos. (C) Lóbulo tiroideo izquierdo, en el corte longitudinal, se observa un nódulo heterogéneo predominantemente hipoecogénico de 2,78 cm (diámetro longitudinal) x 1,5 cm (diámetro transversal). (D) Corte transversal, se observa aumento de volumen y parénquima heterogéneo con algunas áreas hipoecogénicas mal definidas.

Al ingreso a nuestro hospital, la paciente presentaba palpitaciones y tremor en reposo, compatible con signos de tirotoxicosis. Se encontró hormona estimulante de la tiroides (TSH) en 0,043 uUI/mL, elevación de tiroxina libre (T4L) y triyodotironina libre (T3L), anticuerpos antitiroperoxidasa (ATPO) y anticuerpos antitiroglobulina (ATG) negativos, y niveles elevados de tiroglobulina (Tabla 1).

**Tabla 1:** Evolución de los resultados de las pruebas de función tiroidea hasta los 4 meses de seguimiento.

Examen	Ingreso	2 meses	4 meses
TSH (VN: 0,4-4 uUI/mL)	0,043	8,81	2,42
T4L (VN: 0,8-1,9)	4,3	0,95	1,37
T3L (VN: 2,2-5,1)	7,7	--	--
ATPO (U/L)	<10	--	--
ATG (U/L)	<20	--	--
Tiroglobulina (VN: 3-40ng/mL)	118,6	--	24,1

ATG: anticuerpos antitiroglobulina; ATPO: anticuerpos antitiroperoxidasa; T3L: triiodotironina libre; T4L: tiroxina libre; TSH: hormona estimulante de la tiroides; VN: valores normales

La gammagrafía tiroidea con Tecnecio-99m mostró ausencia de captación de radionúclido, compatible con tiroiditis (Figura 2). Debido a las características ecográficas del nódulo del lóbulo tiroideo izquierdo, se realizó biopsia por aspiración con aguja fina (BAAF) guiada por ecografía, siendo su resultado, Bethesda II.

**Figura 2:** Gammagrafía de tiroides con Tecnecio 99 m. (A) y (B) Incidencias anteroposterior. (C) Incidencia oblicua anterior derecha. (D) Incidencia oblicua anterior izquierda. Se muestra captación fisiológica a nivel de las glándulas salivales y ausencia de captación a nivel del lecho tiroideo.

La paciente recibió corticoterapia por dos meses, a base de prednisona, cuya dosis inicial fue 50 mg/día, siendo disminuida de manera progresiva hasta suspenderla. La paciente reportó disminución significativa del dolor una semana después de iniciar la corticoterapia. Al mes, presentaba una frecuencia cardiaca de 70 latidos por minuto y se palpaba la tiroides (bocio grado Ib) con superficie irregular, no dolorosa a la palpación y sin tremor.

A los dos meses, la paciente estaba asintomática, sin corticoides y los resultados de la analítica mostraron TSH 8,81 uUI/mL y T4 libre 0,95 ng/dL. Después de 2 meses más, la paciente permanecía asintomática y se encontró TSH 2,4 UI/mL, T4 libre 1,37 ng/dL, y niveles normales de tiroglobulina (Tabla 1).

Para la elaboración de este artículo, se ha protegido la identidad de la paciente. Contamos con su consentimiento informado y con el permiso del jefe del servicio de endocrinología del Hospital Nacional Edgardo Rebagliati Martins para la publicación.

## DISCUSIÓN

En el 2024, ocurrió un importante brote de dengue en el Perú, que hasta julio había afectado a más de 250,000 personas. Este evento ha resultado en la identificación de numerosos casos con manifestaciones atípicas, como el presente caso ^8^. El caso corresponde a una TSA por dengue, la cual forma parte del síndrome de dengue expandido, una de las manifestaciones inusuales de la infección por dengue severa o estar asociadas con condiciones subyacentes del huésped o coinfecciones ^9^.

La TSA, también conocida como tiroiditis de Quervain, de células gigantes, o granulomatosa, es la causa más frecuente de dolor tiroideo ^10^^,^^11^. Se desarrolla debido a un daño inflamatorio en los folículos tiroideos, que conduce a su destrucción y a la liberación desregulada de hormonas tiroideas ^12^. Se caracteriza por dolor cervical anterior, usualmente acompañado o precedido por fiebre, así como síntomas sistémicos como fatiga, mialgias y artralgias ^12^^-^^14^. El dolor asociado a la TSA puede irradiarse hacia áreas como las zonas parieto-occipitales, orejas, mandíbula, garganta y mediastino superior. La glándula tiroides suele ser palpable, alargada y dolorosa, y en la mayoría de los casos se presentan síntomas leves de tirotoxicosis ^10^^,^^13^^,^^15^.

La TSA tiene una incidencia de dos a cinco casos por cada 100,000 personas por año ^12^ y afecta principalmente a mujeres de entre 25 y 50 años ^14^. Se ha observado asociación con diversas infecciones virales, incluyendo virus de la parotiditis, influenza, rubeola, sarampión, hepatitis E, echovirus, coxsackie, adenovirus, parvovirus B19, ortomixovirus, virus de la inmunodeficiencia humana, Epstein-Barr y SARS-CoV-2, especialmente en individuos genéticamente susceptibles (genotipos HLA-B35 y HLA-B67) en las dos a seis semanas previas al inicio de los síntomas de la TSA ^12^. Aunque su asociación con el virus del dengue no está bien establecida ^16^, en nuestro caso se presentó TSA después de cuatro semanas de la infección por dengue.

La TSA es una condición transitoria y autolimitada que comienza con una fase de tirotoxicosis que dura de tres a seis semanas, en la cual la TSH se encuentra por debajo de 0,01 mU/L, y los niveles de T4L y T3L están elevados o normales ^12^^,^^14^. Esta etapa es seguida por una fase de hipotiroidismo que puede prolongarse hasta seis meses ^14^. Esto último se debe al agotamiento de las reservas y a la falta de síntesis de hormona tiroidea ^15^. En la TSA típicamente se observa la ausencia de anticuerpos antitiroideos. Sin embargo, se ha reportado la presencia de ATPO en 15%, ATG en 30-50%, e incluso anticuerpos contra el receptor de tirotropina (TRAb) en 6%. Esto podría deberse a la liberación de antígenos tiroideos debido al daño glandular, o reflejar una mayor prevalencia de la autoinmunidad tiroidea ^15^

Los hallazgos de nuestro caso se enmarcan en lo descrito en cuanto a las hormonas tiroideas y anticuerpos antitiroideos. Un hallazgo característico en laboratorio es una VSG muy elevada ^14^, además de elevación de la proteína C reactiva, anemia leve, leucocitosis y niveles elevados de tiroglobulina ^10^^,^^14^^,^^15^. En nuestro caso no se pudo solicitar los exámenes de PCR y VSG, información que hubiera contribuido al diagnóstico.

La BAAF de tiroides toma importancia para diferenciar la TSA del cáncer de tiroides o metástasis, ya que comparten características ecográficas, como el caso reportado ^17^. Las características citológicas son inespecíficas y varían según el estadio de la enfermedad. Es común observar gránulos citoplasmáticos intra-vacuolares en células foliculares, granulomas epiteloides y células gigantes multinucleadas en un fondo inflamatorio. Conforme disminuye la inflamación, se observa fibrosis en diferentes grados con granulomas epitelioides ^14^.

La ecografía tiroidea revela áreas hipoecoicas e irregulares ^15^, mientras que la evaluación de la vascularidad mediante Doppler a color muestra flujo normal o disminuido ^12^. Aunque en nuestro caso no se realizó evaluación de la vascularidad, algunos reportes de TSA asociado a dengue mostraron vascularidad aumentada ^16^^,^^18^, lo que podría ser un hallazgo diferencial en el contexto de la infección por dengue. La gammagrafía con Yodo-131 o Tecnecio-99m generalmente muestra una captación baja o nula, tal como se evidenció en nuestra paciente ^12^.

La similitud de sus síntomas con la faringitis puede llevar a un diagnóstico erróneo, resultando en el uso inapropiado de antibióticos en hasta el 50% de los casos ^15^^,^^16^. Además, diferenciar la TSA de la enfermedad de Graves es un reto diagnóstico. No obstante, existen herramientas útiles como el cociente de plaquetas a linfocitos y el cociente de monocitos a eosinófilos, los cuales tienden a ser más elevados en casos de TSA. Se observa un cociente T4 libre/T3 libre mayor a 3,33, así como una relación T4 total/T3 total mayor a 0,041 y un flujo sanguíneo reducido en la arteria tiroidea ^15^^,^^19^. Un diagnóstico erróneo de TSA puede retrasar el reconocimiento de malignidades tiroideas, como las metástasis, o de tiroiditis supurativa, que empeora con la administración de glucocorticoides, y llevar a un tratamiento inadecuado ^14^^,^^17^. No se cuenta con criterios diagnósticos estrictos para TSA, por décadas el diagnóstico se ha basado en un bocio doloroso y aumento en VSG. Sin embargo, Stasiak y colaboradores propusieron criterios diagnósticos que se detallan en la Tabla 2, enfocados en descartar malignidad o confirmar TSA en casos con cuadro clínico poco concluyente como la ausencia de dolor. Describen que para un diagnóstico preciso, se requiere cumplir con los tres criterios principales y al menos uno de los criterios adicionales ^17^.

**Tabla 2:** Criterios diagnósticos de tiroiditis subaguda ^(17)^.

Criterios principales
Elevación de la velocidad de sedimentación globular (o al menos PCR).
Área o áreas hipoecoicas con márgenes borrosos y disminución de vascularización en la ecografía tiroidea.
Resultado de BAAF típico para tiroiditis subaguda o al menos exclusión de malignidad.
Criterios adicionales
Tumefacción dura en la tiroides.
Dolor y sensibilidad en la tiroides.
Elevación de T4L sérico y supresión de TSH.
Reducción de la captación de yodo radioactivo.

BAAF: biopsia por aspiración con aguja fina; PCR: proteína C reactiva; T4L: tiroxina libre; TSH: hormona estimulante de la tiroides; VSG: velocidad de sedimentación globular.

Los beta bloqueadores son útiles para el manejo sintomático, mientras que los antitiroideos no están indicados ^12^^,^^14^. Los antiinflamatorios no esteroideos (AINE) constituyen el tratamiento de primera línea para casos leves a moderados, con los cuales los pacientes suelen mejorar en aproximadamente 5 semanas ^10^^,^^12^^,^^14^. Se considera el uso de glucocorticoides si los síntomas son graves o si el paciente no responde adecuadamente a los AINE ^10^^,^^12^ debido a que proporcionan alivio sintomático en menos de 24 horas ^10^. Típicamente, se recomienda prednisolona o prednisona en dosis de 30-40 mg diarios durante 1-2 semanas, seguidos de una reducción gradual basada a lo largo de un período de seis semanas ^10^^,^^12^. Sin embargo, hasta el 20% de los pacientes pueden requerir tratamiento por más de ocho semanas debido a la reaparición de los síntomas ^10^. En la fase hipotiroidea posterior, puede ser necesario iniciar la reposición con levotiroxina ^14^. Hasta la fecha no existe un protocolo de tratamiento definitivo para la TSA ^10^. Uno de los desafíos en el tratamiento es encontrar un equilibrio entre el riesgo de recurrencia al reducir los corticoides y las complicaciones asociadas con la terapia prolongada ^15^. Aproximadamente el 85-90% de los pacientes recuperan la función tiroidea normal en un período de uno a cuatro meses ^12^^,^^14^. La evolución natural de la TSA no depende de la clínica ni del tratamiento utilizado ^10^.

Se realizó una revisión de los casos publicados hasta la actualidad sobre TSA por dengue, en las bases de datos de Scopus, PubMed y Web of Science, usando como palabras clave: “subacute thyroiditis”, “dengue” y “dengue fever”. Se encontraron seis casos reportados en la literatura ^7^^,^^13^^,^^16^^,^^18^^,^^20^^,^^21^, de los cuales cinco corresponden a varones, con una edad promedio de 35 años (Tabla 3). Esta mayor frecuencia del sexo masculino es diferente a lo descrito en general para la TSA. Desde el inicio de los síntomas del dengue, pasaron en promedio 7,5 días hasta las manifestaciones de la TSA, aunque en nuestro caso el periodo fue 4 veces mayor. En la gran mayoría de los pacientes se observó tumefacción cervical dolorosa, dificultad para la deglución, fiebre, taquicardia y temblores. Solo en dos casos se reportó exoftalmos. La ecografía reveló hallazgos heterogéneos que incluyeron agrandamiento difuso, bocio multinodular y lesión quística sólida compleja, mientras que el Doppler mostró un aumento en la vascularidad. En tres casos se llevó a cabo gammagrafía con Tecnecio-99m que mostró una captación disminuida o ausente, y en un caso se utilizó Iodo-131, con el mismo resultado. Los tratamientos empleados incluyeron corticoides, propranolol, AINE y en un caso, propiltiouracilo debido a la tormenta tiroidea concomitante. Solo se realizó seguimiento en un caso, donde se observó una recuperación de la función tiroidea a las seis semanas.

**Tabla 3:** Casos reportados de TSA asociados a dengue.

Autor, país, año	Sexo	Edad (años)	Tiempo (días) *	Síntomas	VSG (mm/h)	TSH (UI/mL)	Ecografía	Gammagrafía	Tratamiento
Mangaraj, India, 2021	F	38	10	Tumefacción cervical dolorosa, fiebre, taquicardia, tremor	86	0,06	Alargamiento difuso, parénquima heterogéneo. Vascularidad: leve incremento	No captación de 99mTc	Propranolol, prednisolona
Bhushan, India, 2018	M	23	5	Tumefacción cervical dolorosa, taquicardia, exoftalmos	101	0,17	Lesión quística, sólica, compleja en lóbulo derecho. Vascularidad: incrementada	Poca captación de 99mTc	Propanolol, esteroides, AINE, antibióticos
Dwipayana, Indonesia, 2017	M	33	--	Tumefacción cervical, fiebre, taquicardia, exoftalmos, tremor	--	0,05	Bocio multinodular bilateral	--	Propranolol, pulso EV de metilprednisolona, ceftriaxona, propiltiouracilo
Mo Z, China, 2016	M	65	6	No especifíca	--	0,09	--	--	Pulso EV de metilprednisolona
Sheraz, Pakistán, 2016	M	32	--	Tumefacción cervical dolorosa, dificultad para deglución, fiebre, taquicardia, tremor	43	0,05	--	Poca captación de I131	Propranolol, esteroides, ibuprofeno
Assir, Pakistán, 2012	M	20	9	Tumefacción cervical dolorosa, fiebre, taquicardia, tremor	62	0,01	Vascularidad: Disminuida	No captación de 99mTc	Propranolol, prednisona

* Tiempo se refiere al intervalo transcurrido entre el inicio de los síntomas del dengue y el inicio de los síntomas de la TSA. 99mTc: gammagrafía con Tecnecio-99m; AINE: antiinflamatorios no esteroideos: EV: endovenoso; F: femenino; I131: gammagrafía con Yodo-131; M: masculino; TSH: hormona estimulante de la tiroides; VSG: velocidad de sedimentación globular.

Las limitaciones de nuestro estudio incluyeron la falta de evaluación de la vascularidad tiroidea mediante Doppler y la ausencia de medición de la VSG. No obstante, los análisis hormonales y la gammagrafía confirmaron el diagnóstico.

En conclusión, nuestro reporte subraya la importancia de identificar las manifestaciones atípicas del dengue, como la TSA, la cual es poco frecuente y su diagnóstico requiere un alto grado de sospecha, teniendo en cuenta la clínica, el laboratorio y el antecedente epidemiológico, dado que la gammagrafía tiroidea no siempre está disponible en todos los medios. En los últimos años para el diagnóstico toma importancia también la ecografía y la BAAF de tiroides cuando hay duda en el diagnóstico.
